# Behavioral and neural interaction between spatial inhibition of return and the Simon effect

**DOI:** 10.3389/fnhum.2013.00572

**Published:** 2013-09-17

**Authors:** Pengfei Wang, Luis J. Fuentes, Ana B. Vivas, Qi Chen

**Affiliations:** ^1^Center for Studies of Psychological Application and School of Psychology, South China Normal UniversityGuangzhou, China; ^2^Departamento de Psicología Básica y Metodología, Facultad de Psicología, Universidad de MurciaMurcia, Spain; ^3^Psychology Department, The University of Sheffield International FacultyCity College, Greece

**Keywords:** spatial IOR, the Simon effect, fMRI, shared spatial representation, parietal cortex, frontal cortex

## Abstract

It has been well documented that the anatomically independent attention networks in the human brain interact functionally to achieve goal-directed behaviors. By combining spatial inhibition of return (IOR) which implicates the orienting network with some executive function tasks (e.g., the Stroop and the flanker tasks) which implicate the executive network, researchers consistently found that the interference effects are significantly reduced at cued compared to uncued locations, indicating the functional interaction between the two attention networks. However, a unique, but consistent effect is observed when spatial IOR is combined with the Simon effect: the Simon effect is significantly larger at the cued than uncued locations. To investigate the neural substrates underlying this phenomenon, we orthogonally combined the spatial IOR with the Simon effect in the present event-related fMRI study. Our behavioral data replicated previous results by showing larger Simon effect at the cued location. At the neural level, we found shared spatial representation system between spatial IOR and the Simon effect in bilateral posterior parietal cortex (PPC); spatial IOR specifically activated bilateral superior parietal cortex while the Simon effect specifically activated bilateral middle frontal cortex. Moreover, left precentral gyrus was involved in the neural interaction between spatial IOR and the Simon effect by showing significantly higher neural activity in the “Cued_Congruent” condition. Taken together, our results suggest that due to the shared spatial representation system in the PPC, responses were significantly facilitated when spatial IOR and the Simon effect relied on the same spatial representations, i.e., in the “Cued_Congruent” condition. Correspondingly, the sensorimotor system was significantly involved in the “Cued_Congruent” condition to fasten the responses, which indirectly resulted in the enhanced Simon effect at the cued location.

## Introduction

It is amply accepted that there exist three functionally and anatomically independent attention networks in the human brain: the alerting network, the orienting network and the executive network (Petersen et al., 1989; Posner and Petersen, 1990; Fan et al., 2002, 2003b, 2005, 2009). The alerting network provides the ability to increase vigilance to an impending stimulus. This network consists of thalamic and some specific anterior and posterior cortical sites, and involves the cortical projection of the norepinephrine system (Fan et al., 2005, 2009; Federico et al., 2013). The orienting network is responsible for reflexively or voluntarily shifting visuospatial attention to a specific location to sample sensory input (Corbetta et al., 2000; Yantis et al., 2002; Fan et al., 2005; Kincade et al., 2005). For example, the orienting network is involved in a spatial inhibitory mechanism that prevents the attention system from re-examining previously attended locations. This mechanism was first described in the Posner's spatial cuing task, in which a peripheral cue was first presented to attract spatial attention to the cue location (Posner and Cohen, 1984; Posner et al., 1985; Klein, 2000). Responses to a target immediately appearing at the cued location, compared to responses to a target at an uncued location, were both faster and more accurate. However, if the cue-target stimulus onset asynchrony (SOA) was longer than 300 ms and the cue was uninformative with regard to target location, responses to the target at the cued location would be delayed, compared to responses to the target at the uncued location. This inhibitory effect is termed inhibition of return (IOR) (Posner and Cohen, 1984), which slows down attentional reorienting to the previously attended (cued) location, and thus increases the efficiency of visual search (Zhou and Chen, 2008; McDonald et al., 2009; Tian et al., 2011). Neurally, a dorsal frontoparietal network, including bilateral frontal eye field (FEF), the superior and inferior parietal cortex, are involved in the orienting network (Rosen et al., 1999; Corbetta and Shulman, 2002; Mayer et al., 2004; Zhou and Chen, 2008; Fan et al., 2009). The executive network manages the ability to control behavior to achieve intended goals and resolve conflict among alternative responses (Posner and Petersen, 1990). It has been generally measured by the Stroop task, the flanker task, and the Simon task (Umiltá and Nicoletti, 1990; Lu and Proctor, 1995; Botvinick et al., 2001; Fan et al., 2009). At the neural level, the executive function has been associated with anterior cingulate cortex (ACC) and lateral prefrontal cortex (MacDonald et al., 2000; Fan et al., 2005, 2009; Zhou et al., 2011).

Although there has been extensive evidence suggesting the functional and anatomical independences between the executive and the orienting networks, the attention networks need to interact in multiple ways to achieve coherent, goal-directed behaviors (Fuentes, 2004; Fuentes et al., 2012). For example, at the behavioral level, when the Stroop or flanker interference tasks are combined in the manipulation of IOR such that conflicting information can be presented at either the cued or the uncued location, the interference effects are reduced, eliminated or even reversed at the cued location (Fuentes et al., 1999; Vivas and Fuentes, 2001; Vivas et al., 2007). At the neural level, when spatial IOR, a mechanism associated with the orienting network, was orthogonally combined with non-spatial IOR, a mechanism associated with the executive network, the orienting and the executive networks interacted and compensated each other in biasing the attention system for novelty (Chen et al., 2010). The orienting network was involved in slowing down responses to the old location only when the non-spatial IOR mechanism in the executive network was not operative (i.e., when the non-spatial feature of the target was novel); the prefrontal executive network was involved in slowing down responses to the old non-spatial representation only when the spatial IOR mechanism in the orienting network was not functioning (i.e., when the target appeared at a novel location).

One exceptional case to the above findings, however, is when spatial IOR is combined with the Simon effect. Although previous studies found that the Stroop and the flanker conflicts were reduced or even reversed at the inhibited (cued) location of spatial IOR, an effect attributed to an executive-dependent inhibitory tagging mechanism (Fuentes et al., 1999, 2012; Vivas and Fuentes, 2001; Fuentes, 2004), the Simon conflicts were significantly increased at the cued location (Lupiáñez, Milán, Tornay, Madrid and Tudela, 1997; Pratt et al., 1997; Ivanoff et al., 2002; Hilchey et al., 2011). The Simon effect refers to the phenomenon that even when the spatial location of stimuli is task-irrelevant, participants' responses are slower when the spatial location of the stimuli is contralateral to the predefined location of response (i.e., incongruent condition) than when they are ipsilateral (i.e., congruent condition) (Umiltá and Nicoletti, 1990; Lu and Proctor, 1995). For example, in a color discrimination task, one of two color stimuli is presented either on the left or right side of the computer screen, and participants are instructed to press the left-side key in response to one color and to press the right side key in response to the other color. Although the spatial location of the color stimulus is irrelevant concerning the color discrimination task, participants' responses are slower when the spatial position of the color stimulus (left or right) was contralateral to the position of the response key (left or right) than when they are ipsilateral.

The “amplification of the Simon effect by IOR” can be interpreted as the consequence of the inhibitory mechanism: when stimuli fell at inhibited (cued) locations, access to the response system from the task-relevant dimension of the target (e.g., color) was hindered such that the competition from the task-irrelevant dimension of the target (e.g., location) was increased. Therefore, the increased Simon effect at the cued location due to the incongruent condition being affected at that inhibited location. Another similar but slightly different interpretation is that, IOR delayed both codes activated by the target (the task-relevant identity code and the task-irrelevant location code). However, the delaying effect of IOR on spatial processing (localization) was much greater than it is on non-spatial processing (Hilchey et al., 2011), so that the responses in the “Cued_Incongruent” condition were significantly delayed, compared with the “Cued_Congruent” condition.

However, an alternative hypothesis, the shared spatial representation account, cannot be rejected. In contrast to the Stroop effect and the flanker effect, in which the conflicts are induced between two non-spatial semantic representations, the conflicts in the Simon effect are between the response-related spatial representation activated by the task-relevant dimension of the target (e.g., color) and the response-related spatial representation activated by the task-irrelevant dimension (e.g., location) of the target. Therefore, if the Simon task is combined with the spatial IOR task, the shared spatial representation system could be activated when the aforementioned spatial representations coincide, especially when a congruent stimulus is presented at the cued location. Specifically speaking, when the target appears at the cued location and the cued location is on the same side as the response key required by the target, the shared spatial representation between the cued location of spatial IOR and the position of the response key in the Simon task could cause significantly faster responses (i.e., a facilitatory effect) in the “Cued_Congruent” condition compared with the “Cued_Incongruent” condition, indirectly resulting in the increased size of the Simon effect observed at the cued location.

In the present event-related fMRI study, we orthogonally combined the spatial IOR procedure with the Simon task. We aimed to investigate the neural correlates of the Simon effect and spatial IOR, and explore the neural substrates underlying the increased size of the Simon effect at the cued location by examining the two alternative hypotheses, the inhibitory hypothesis and the shared spatial representation hypothesis. If the inhibitory hypothesis is correct, we should expect higher prefrontal areas activation in the incongruent than in the congruent condition at the cued location, in comparison with neural activations at the uncued location. If the shared spatial representation hypothesis is correct, we predict that we will find shared spatial representations areas [e.g., the posterior parietal cortex (PPC), (Haxby et al., 1991; Sack, 2009)] between the spatial task and the conflict task. In addition, we should find higher neural activation in the congruent than the incongruent conditions at the cued location, in comparison with neural activation at the uncued location.

## Materials and methods

### Participants

Sixteen undergraduate students (9 males and 7 females, 24 ± 3 years old) participated in the present study. They were all right handed and had normal or corrected-to-normal visual acuity. None of them had a history of neurological or psychiatric disorders. All participants gave informed consent prior to the experiment in accordance with the Helsinki declaration, and the study was approved by the ethics committee of the School of Psychology, South China Normal University.

### Stimuli and experimental design

**Figure 1:**
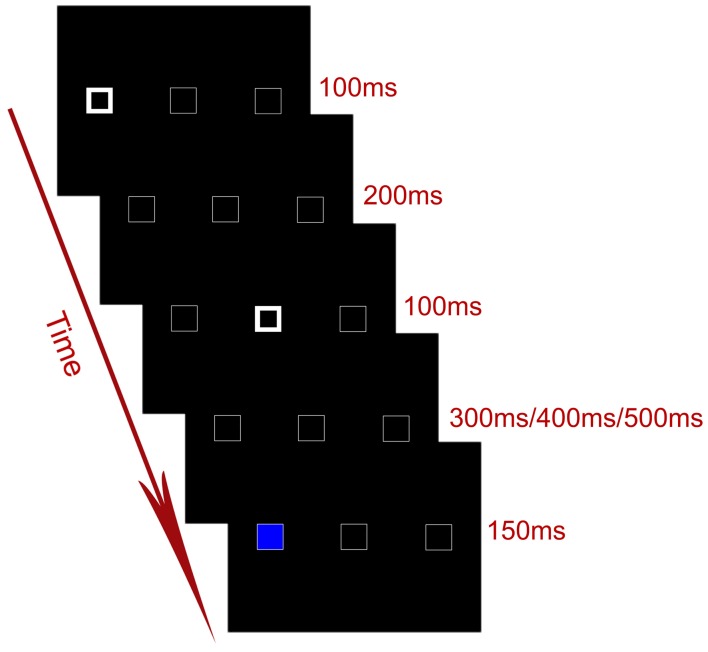
**Timing of an exemplar trial in the experiment**.

The stimuli were presented through a LCD projector onto a rear projection screen located behind the participants' head. Participants viewed the screen through an angled mirror on the head-coil. Each trial consisted of a serial of displays (Figure 1). The default display included three horizontally arranged white boxes (1.9° × 1.9° visual angle) on a black background. The center-to-center distance between two adjacent boxes was 7.4° in visual angle. Participants were instructed to fixate at the central box throughout the experiment. At the beginning of each trial, the outlines of one of the two peripheral boxes became thicker and brighter for 100 ms, serving as a cue to attract spatial attention to one of the peripheral locations. The cue was uninformative with regard to the location of the target, i.e., the target appeared at the cued location in 50% of the total trials. After an interval of 200 ms, the outlines of the central box became thicker for 100 ms, serving as a central cue to attract attention from the cued peripheral location to the center. After another interval of 300, 400, or 500 ms, the target (a blue or yellow patch) appeared in either the cued or the uncued peripheral box for 150 ms. Note that the purpose of using variable cue–target SOAs was to prevent participants from forming time-based expectations for the target.

While lying in the scanner, participants hold a response pad in each of their two hands, and the two response pads were positioned on the left and right side of the body. The behavioral task was to discriminate the color of the target, irrespective of the location of the target. Participants were instructed to press one button with the thumb of one hand if the color of the target was blue, and the other button with the thumb of the other hand if the color of the target was yellow. The mapping between the two response hands and the color of the target was counterbalanced across participants. The spatial location of the target, though irrelevant to the color discrimination task, could be either congruent (i.e., ipsilateral) or incongruent (i.e., contralateral) with the side of the response hand.

Therefore, the present experimental design was a 2 (cue validity: cued vs. uncued) ×2 (Simon congruency: congruent vs. incongruent) event-related fMRI factorial design. There were four experimental conditions in the factorial design and 48 trials for each condition. In total, there were 256 trials, consisting of 192 experimental trials and 64 null trials. In the null trials, only the default display was presented. The inter-trial intervals (ITIs) were jittered from 2200 to 3200 ms (2200, 2450, 2700, 2950, and 3200 ms) with a mean ITI of 2700 ms. All participants completed a training section of 6 min outside the scanner before the scanning.

### Statistical analysis of behavioral data

Incorrect responses and RTs longer than mean RT plus three times standard deviation (SD) or shorter than mean RT minus three times SD were excluded from further analysis. Mean RTs and error rates were then calculated and submitted to a 2 (cue validity: cued vs. uncued) ×2 (Simon congruency: congruent vs. incongruent) repeated-measures ANOVA. Significant effects were further examined by planned *t* tests.

### Data acquisition and pre-processing

A 3T Siemens Trio system with a standard head coil (Erlangen, Germany) was used to obtain T2*-weighted echo-planar images (EPI) with blood oxygenation level-dependent (BOLD) contrast (matrix size: 64 × 64, voxel size: 3.1 × 3.1 × 3.0 mm^3^). Thirty-six transversal slices of 3 mm thickness that covered the whole brain were acquired sequentially with a 0.3 mm gap (*TR* = 2.2 s, *TE* = 30 ms, *FOV* = 220 mm, flip angle = 90°). The one-run functional scanning had 330 EPI volumes, and the first five volumes were discarded to allow for T1 equilibration effects.

Data were pre-processed with Statistical Parametric Mapping software SPM8 (Wellcome Department of Imaging Neuroscience, London, http://www.fil.ion.ucl.ac.uk). Images were realigned to the first volume to correct for inter-scan head movements. Then, the mean EPI image of each subject was computed and spatially normalized to the MNI single subject template using the “unified segmentation” function in SPM8. This algorithm is based on a probabilistic framework that enables image registration, tissue classification, and bias correction to be combined within the same generative model. The resulting parameters of a discrete cosine transform, which define the deformation field necessary to move individual data into the space of the MNI tissue probability maps, were then combined with the deformation field transforming between the latter and the MNI single subject template. The ensuing deformation was subsequently applied to individual EPI volumes. All images were thus transformed into standard MNI space and re-sampled to 2 × 2 × 2 mm^3^ voxel size. The data were then smoothed with a Gaussian kernel of 8 mm full-width half-maximum to accommodate inter-subject anatomical variability.

### Statistical analysis of imaging data

Data were high-pass-filtered at 1/128 Hz and were then analyzed with a general linear model (GLM) as implemented in SPM8. Temporal autocorrelation was modeled using an AR (1) process. At the individual level, the GLM was used to construct a multiple regression design matrix that included four experimental events: (1) the target appeared at the cued location, and its response hand was ipsilateral to its location (Cued_Congruent); (2) the target appeared at the cued location, and its response hand was contralateral to its location (Cued_Incongruent); (3) the target appeared at the uncued location, and its response hand was ipsilateral to its location (Uncued_Congruent); (4) the target appeared at the uncued location, and its response hand was contralateral to its location (Uncued_Incongruent). The four events were time-locked to the target of each trial by a canonical synthetic hemodynamic response function (HRF) and its temporal and dispersion derivatives, with event duration of 0 s. The inclusion of the dispersion derivatives took into account the different durations of neural processes induced by the variable cue–target intervals and allowed for changes in dispersion of the BOLD responses induced by different cue–target intervals. Additionally, all the instructions, omissions, error trials were separately modeled as regressors of no interest. Parameter estimates were subsequently calculated for each voxel using weighted least-squares to provide maximum likelihood estimators based on the temporal autocorrelation of the data. No global scaling was applied.

For each participant, simple main effects for each of the four experimental conditions were computed by applying appropriate baseline contrasts [i.e., the experimental conditions vs. implicit baseline (null trials) contrasts]. The four first-level individual contrast images were then fed into a 2 × 2 within-participants ANOVA at the second group level employing a random-effects model (the flexible factorial design in SPM8 including an additional factor modeling the subject means). In the modeling of variance components, we allowed for violations of sphericity by modeling non-independence across parameter estimates from the same subject, and allowed for unequal variances between conditions and between subjects using the standard implementation in SPM8. Areas of activation in the main effects and the interaction effects were identified as significant only if they passed a conservative threshold of *P* < 0.001, corrected for multiple comparisons at the cluster level with an underlying voxel level of *P* < 0.001, uncorrected (Poline et al., 1997).

## Results

### Behavioral data

**Figure 2:**
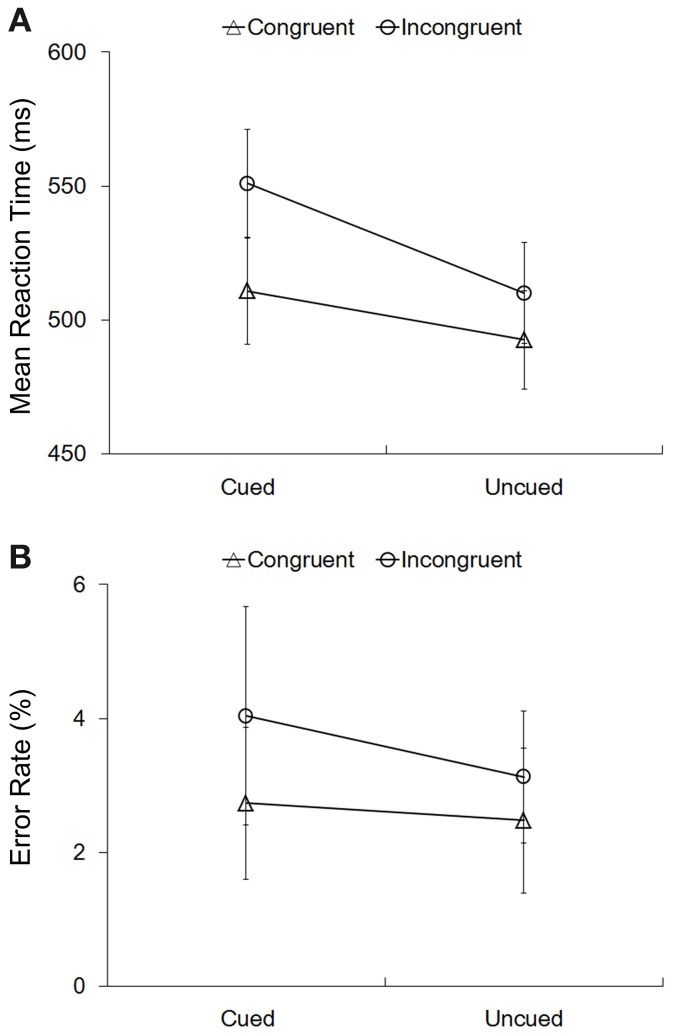
**Behavioral results.** Mean RTs **(A)** and error rates **(B)** in the four experimental conditions. The error bars showed the standard errors of mean RTs **(A)** and error rates **(B)**.

Mean RTs in the four experimental conditions were submitted to a 2 (cue validity: cued vs. uncued) ×2 (Simon congruency: congruent vs. incongruent) repeated-measures ANOVA (Figure 2A). The main effect of cue validity was significant, *F*_(1, 15)_ = 31.91, *p* < 0.001, η^2^ = 0.68, indicating that RTs to the cued targets (531 ± 20 ms) were significantly slower than RTs to the uncued targets (501 ± 18 ms), i.e., a significant IOR effect. The main effect of Simon congruency was also significant, *F*_(1, 15)_ = 22.31, *p* < 0.001, η^2^ = 0.60, indicating that RTs in the congruent condition (502 ± 19 ms) were significantly faster than RTs in the incongruent condition (530 ± 19 ms), i.e., a significant Simon effect. More importantly, the interaction between cue validity and the Simon congruency was significant, *F*_(1, 15)_ = 10.15, *p* = 0.006, η^2^ = 0.40 (Figure 2A). Planned paired *t*-tests on simple effects further showed that, on the one hand, the size of the Simon effect was significantly larger at the cued location (40 ± 30 ms) than at the uncued location (17 ± 26 ms), *t*_(15)_ = 3.186, *p* = 0.006. On the other hand, the size of IOR was significant larger in the incongruent condition (41 ± 27) than in the congruent condition (18 ± 23), *t*_(15)_ = 3.186, *p* = 0.006. The error rates (Figure 2B) had the same pattern as the RTs, but further 2 × 2 repeated-measures ANOVA showed that, neither the main effects of the cue validity and the Simon congruency nor the interaction effect were significant (all *p* > 0.1).

### Imaging data

#### Common and specific neural correlates underlying spatial IOR and the simon effect

**Figure 3:**
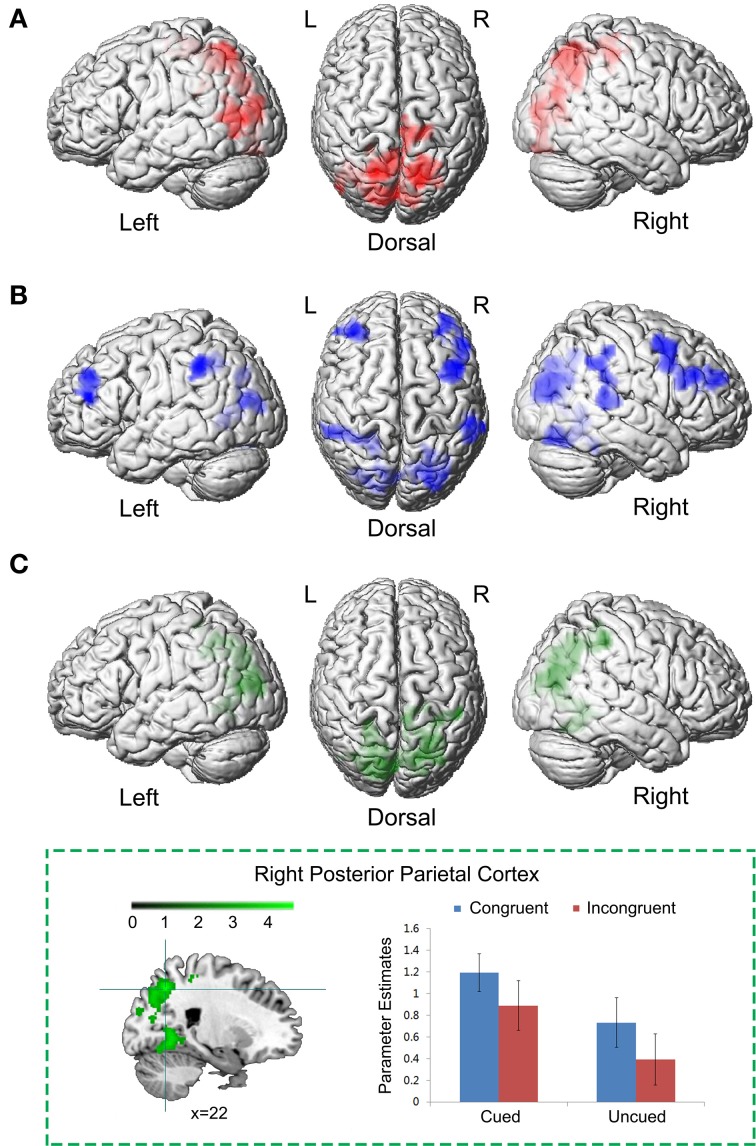
**Common neural correlates underlying spatial IOR and the Simon effect. (A)** Brain regions associated with the cue validity, i.e., the main effect contrast “Cued (Congruent + Incongruent) > Uncued (Congruent + Incongruent).” **(B)** Brain regions associated with the Simon effect, i.e., the main effect contrast “Congruent (Cued + Uncued) > Incongruent (Cued + Uncued).” **(C)** The conjunction analysis between **(A)** and **(B)**.

We first identified brain regions associated with the cue validity of spatial IOR. Right PPC, extending inferior to right middle occipital cortex and superior to bilateral superior parietal cortex, showed significantly higher neural activity to targets at the cued location than uncued location, i.e., the main effect contrast “Cued (Congruent + Incongruent) > Uncued (Congruent + Incongruent)” (Figure 3A; Table 1A). No significant activation was found in the reverse contrast. We then calculated the brain regions activated by the main effect of the Simon congruency. Bilateral inferior frontal gyrus, bilateral middle occipital gyrus extending to right superior occipital cortex and left superior parietal cortex, and right middle temporal cortex showed significantly higher neural activity in the congruent condition than in the incongruent condition, i.e., the main effect contrast “Congruent (Cued + Uncued) > Incongruent (Cued + Uncued)” (Figure 3B; Table 1B). No significant activation was found in the reverse contrast.

**Table 1:** **Brain regions showing significant relative increases of BOLD response associated with the cue validity (cued vs. uncued) and the Simon congruency (congruent vs. incongruent)**.

**Anatomical region**	**Side**	**Cluster peak (mm)**	***t*-Score**	**kE (voxels)**
**A CUED > UNCUED**				
Superior parietal gyrus	R	20, −62, 46	6.04	5477
*Middle occipital gyrus*	*L*	−*34*, −*72*, *16*	*5.85*	
*Superior parietal gyrus*	*L*	−*18*, −*64*, *64*	*5.16*	
**B CONGRUENT > INCONGRUENT**				
Middle frontal gyrus	R	52, 26, 34	6.65	1226
*Inferior frontal gyrus*	*R*	*50*, *20*, *32*	*5.67*	
Fusiform	R	26, −78, −10	5.42	793
Middle frontal gyrus	L	−34, 34, 20	5.16	499
*Inferior frontal gyrus*	*L*	−*52*, *36*, *22*	*4.51*	
Superior occipital gyrus	R	24, −80, 22	4.95	2598
*Middle occipital gyrus*	*L*	−*28*, −*80*, *18*	*4.92*	
*Middle occipital gyrus*	*R*	*28*, −*78*, *26*	*4.92*	
Superior parietal gyrus	L	−18, −56, 46	4.91	698
Middle temporal gyrus	R	64, −40, 12	4.04	634
**C (CUED > UNCUED) ∩ (CONGRUENT > INCONGRUENT)**				
Posterior parietal cortex	R	22, −62, 46	4.60	4936
*Middle occipital gyrus*	*L*	−*22*, −*90*, *16*	*4.45*	
*Middle occipital gyrus*	*R*	*30*, −*78*, *30*	*4.24*	
*Posterior parietal cortex*	*L*	−*22*, −*60*, *50*	*3.62*	

*The coordinates (x, y, z) correspond to MNI coordinates. Displayed are the coordinates of the maximally activated voxel within a significant cluster as well as the coordinates of relevant local maxima within the cluster (in italics)*.

Since the neural network involved in the main effect of spatial cue validity (Figure 3A) and the neural network involved in the main effect of the Simon congruency (Figure 3B) partly overlapped, in order to isolate the common and specific neural correlates underlying the two main effects, we further performed a conjunction analysis and an exclusive masking procedure between them. The conjunction analysis between the main effect of spatial cue validity (cued > uncued), and the main effect of Simon congruency (congruent > incongruent) showed significant activations in bilateral PPC extending to bilateral middle occipital gyrus (Figure 3C; Table 1C).

**Figure 4:**
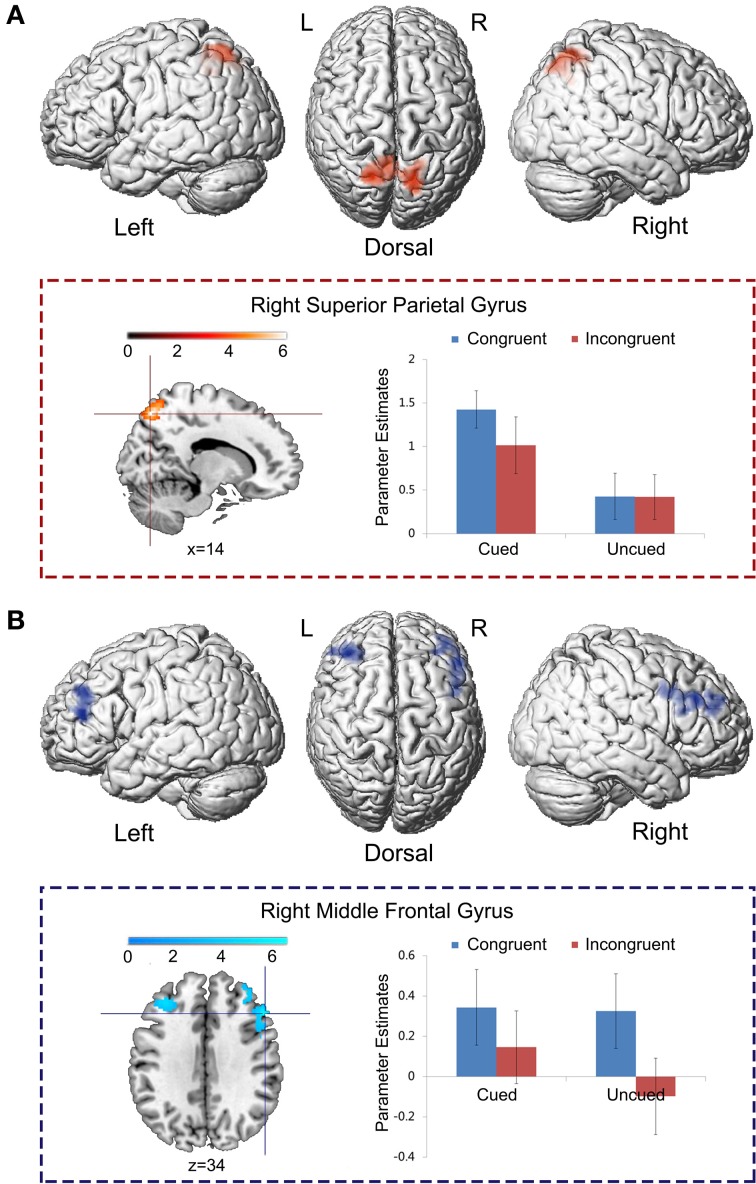
**Specific neural correlates underlying spatial IOR and the Simon effect. (A)** The main effect contrast “Cued > Uncued” was exclusively masked by the mask contrast “Congruent > Incongruent” at the threshold of *p* < 0.05, uncorrected for multiple comparisons. **(B)** The main effect contrast “Congruent > Incongruent” was exclusively masked by the mask contrast “Cued > Uncued” at the threshold of *p* < 0.05, uncorrected for multiple comparisons.

To isolate the brain regions that were significantly involved in the main effect of spatial cue validity, but not in the main effect of the Simon congruency, the main effect contrast “Cued > Uncued” was exclusively masked by the mask contrast “Congruent > Incongruent” at a liberal threshold of *p* < 0.05, uncorrected for multiple comparisons. In this way, those voxels that reached a level of significance at *p* < 0.05 (uncorrected) in the mask contrast were excluded from the analysis. Bilateral superior parietal cortex was exclusively involved in the main effect of spatial cue validity, rather than the main effect of Simon congruency (Figure 4A; Table 2A).

**Table 2:** **Brain regions showing significant relative increases of BOLD response associated with the cue validity (cued vs. uncued) and the Simon congruency (congruent vs. incongruent)**.

**Anatomical region**	**Side**	**Cluster peak (mm)**	***t*-score**	**kE (voxels)**
**A (CUED > UNCUED) MASKED BY (CONGRUENT > INCONGRUENT)**				
Superior parietal gyrus	R	14, −72, 54	5.16	940
*Superior parietal gyrus*	*L*	−*18*, −*64*, *64*	*5.16*	
**B (CONGRUENT > INCONGRUENT) MASKED BY (CUED > UNCUED)**				
Middle frontal gyrus	R	52, 26, 34	6.65	488
Middle frontal gyrus	L	−34, 34, 20	5.16	435

*The coordinates (x, y, z) correspond to MNI coordinates. Displayed are the coordinates of the maximally activated voxel within a significant cluster as well as the coordinates of relevant local maxima within the cluster (in italics)*.

To isolate the brain regions which were involved only in the main effect of the Simon congruency, but not in the main effect of cue validity, the main effect contrast “Congruent > Incongruent” was exclusively masked by the mask contrast “Cued > Uncued.” Bilateral middle frontal gyrus was exclusively activated by the main effect of the Simon congruency, rather than by the main effect of cue validity (Figure 4B; Table 2B).

#### Neural interaction between spatial IOR and the simon effect

**Figure 5:**
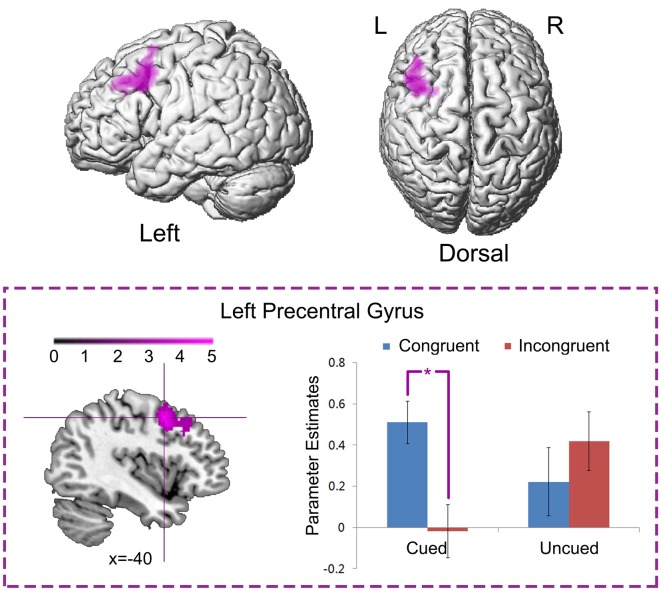
**Neural interaction between spatial IOR and the Simon effect.** Left precentral gyrus was significantly activated by the neural interaction contrast “Cued (Congruent > Incongruent) > Uncued (Congruent > Incongruent).” Parameter estimates in the four experimental conditions were extracted from the activated cluster, and are displayed as a function of the experimental conditions (^*^, *P* < 0.001).

Left precentral gyrus (MNI: −40, 6, 48; *t* = 5.30, 576 voxels) was significantly activated by the neural interaction contrast “Cued (Congruent > Incongruent) > Uncued (Congruent > Incongruent)” (Figure 5). Parameter estimates in the four experimental conditions were extracted from the activated cluster. Planned paired *t*-tests on simple effects suggested that neural activity was significantly increased in the congruent condition compared to the incongruent conditions when the targets appeared at the cued location, *t*_(15)_ = 5.91, *p* < 0.001, while there was no significant difference between the congruent and incongruent conditions when the targets appeared at the uncued location, *p* > 0.1. No significant activation was found in the reverse interaction contrast.

## Discussion

In the present fMRI study, we aimed to further investigate the interactions between spatial inhibitory processes, indexed by the spatial-based IOR phenomenon, and response-based conflict processes, indexed by the Simon task. Our previous research showed that inhibitory mechanisms triggered by presenting conflicting stimuli at locations subject to spatial IOR, caused striking patterns of interactions. Concretely, Stroop and flanker interference effects were reduced, eliminated or even reversed at the cued (inhibited) location (for recent reviews, see Fuentes, 2004; Fuentes et al., 2012). However, contrary to the Stroop and flanker tasks, the Simon and the IOR procedures activate response-related spatial representations, which might be responsible for the specific pattern of interactions observed when both procedures are combined in a single experiment: the Simon interference effect is increased when stimuli are presented at the cued location (see Ivanoff et al., 2002; Hilchey et al., 2011). We replicated that pattern of interaction at the behavioral level by showing increased size of Simon effects at the cued locations. This phenomenon might occur either by the delayed responses in the “Cued_Incongruent” condition (inhibitory hypothesis), or by the facilitated responses in the “Cued_Congruent” condition (shared spatial representation hypothesis). Our neural data supported the latter interpretation and revealed the neural mechanisms of the interaction between the Simon and IOR effect.

Regarding the behavioral data, previous researches have reported Simon congruency effects of around 20 ms size (De Jong et al., 1994; Vallesi et al., 2005; Nishimura and Yokosawa, 2010), and spatial IOR of around 40 ms size (Posner and Cohen, 1984; Klein, 2000) when each task is used in isolation. In our combined procedure, we report 17 ms for Simon congruency and 41 ms for IOR in the uncued location (Uncued_Incongruent > Uncued_Congruent) and the incongruent condition (Cued_Incongruent > Uncued_Incongruent), respectively. Thus, uncued locations and incongruent stimuli, the combined conditions that do not share any spatial representation, behaved as the standard conditions for each task, producing effect sizes in the standard range. Importantly, IOR reduced up to 18 ms as a consequence of response facilitation in the congruent trials when presented at the cued location (Cued_Congruent > Uncued_Congruent), which concurrently produced an increase in the Simon effect up to 40 ms (Cued_Incongruent > Cued_Congruent). Briefly, it seemed that the facilitated responses in the Cued_Congruent condition, rather than delayed responses in the Cued_Incongruent condition produced the significant interaction between spatial IOR and the Simon effect. Note, that in the present study we didn't make a further comparison of both conditions to a neutral condition, and then a direct assessment of whether the aforementioned interaction pattern is better accounted for in terms of facilitation or inhibition is not possible. However, on the basis of the effect sizes observed in both cued-uncued locations and congruent-incongruent conditions, our present results clearly support the shared spatial representation hypothesis. This is further supported by the neural results, as we will discuss later on. The ocular-motor theory of IOR emphasizes the correlation between IOR and oculomotor system: the peripheral cue produces an automatic activation of an eye movement to that location, which generates IOR (Rafal et al., 1989; Kingstone and Pratt, 1999; Klein, 2000). And, both spatial IOR and the Simon effect could be influenced by eye movements (Abrahamse and Van der Lubbe, 2008; Buetti and Kerzel, 2010; Khalid and Ansorge, 2013). In the present study, in order to minimize the effects of eye movements, we instructed the participants to fixate at the central box throughout the experiment. Due to technical limitations, however, we couldn't track the eye movements during the fMRI-scanning.

Regarding neural data, we replicated brain activations that had been associated with either spatial IOR or Simon effects. Spatial IOR specifically activated the bilateral superior parietal cortex (Figure 4A). This finding was consistent with prior ERP and fMRI studies on spatial IOR (Zhou and Chen, 2008; Tian et al., 2011). Within the dorsal frontoparietal network, the bilateral superior parietal cortex plays an important role in voluntarily/involuntarily orienting visuospatial attention between spatial representations of external locations (Ungerleider and Mishkin, 1982; Goodale and Milner, 1992). For example, neuropsychological studies have shown that patients with superior parietal lesions were impaired in detecting the displacement of a visual stimulus and showed erratic fixation pattern in attention tasks (Phan et al., 2000; Vandenberghe et al., 2012). Neuroimaging studies with healthy adults further showed that the activity of the superior parietal cortex exhibited transient enhancement when attention was shifted between spatial locations [refer to Behrmann et al. (2004)].

On the other hand, the Simon task specifically activated the bilateral middle frontal cortex (Figure 4B). Previous neuroimaging studies suggest that compared to the congruent condition, a frontoparietal network is activated in the incongruent condition (Maclin et al., 2001; Fan et al., 2003a; Liu et al., 2004). For example, in the Liu et al. (2004)'s study, an arrow pointing upwards or downwards was presented on the left or right side of a central fixation point. Participants responded to one arrow with the index finger (left-most) and to the other with the middle finger (right-most) of their right hand. The incongruent condition, compared to the congruent condition, significantly activated the ACC, the dorsolateral prefrontal cortex (DLPFC), the precuneus, and the pre-supplementary motor area (pre-SMA). According to the conflict-monitoring theory (Botvinick et al., 1999, 2001, 2004) the ACC is responsible for monitoring conflict and response errors, whereas the DLPFC, which receives signals from the ACC, would be involved in modulating processing in the PPC by biasing the system toward the task-relevant information.

Our conjunction results provide unequivocal support for the shared spatial representation theory. The conjunction analysis between the two main effect contrasts “Cued > Uncued” and “Congruent > Incongruent” showed that the PPC in both hemispheres is the common site responsible of the interaction between IOR and the Simon effect (Figure 3C). The PPC is part of the dorsal visual stream involved in coding the spatial location of a stimulus (“where”), in contrast to the ventral visual stream, which is mainly devoted to the perceptual identification of objects (“what”) (Ungerleider and Mishkin, 1982; Goodale et al., 1991; Goodale and Milner, 1992; Milner and Goodale, 1995, 2008). A large body of brain imaging literature points to a particular role for the PPC in multiple space representations (Kesner, 2009; Sack, 2009) and spatial cognition (Haxby et al., 1991; Colby and Goldberg, 1999; Landis, 2000; Marshall and Fink, 2001; Sack, 2009). For example, Andersen et al. (1997) argued that by using a specific gain mechanism, the PPC may combine different coordinated frames coming from various input spatial signals into common distributed spatial representations. Previous Transcranial Magnetic Stimulation (TMS) studies have clearly shown that, the PPC plays a crucial role in spatial representation both in the IOR and the Simon task. For example, TMS over areas of the right PPC has proven able to disrupt manual IOR (Chica et al., 2011), and IOR spatial remapping (van Koningsbruggen et al., 2010). Similarly, TMS over areas of the right PPC produced a reduction of the Simon effect (Schiff et al., 2008). In these studies, the results were interpreted as the disruption of the spatial representation. In the present study, both spatial IOR and the Simon task activated spatial representations from left and right locations. In spatial IOR, visuospatial attention is oriented/reoriented between spatial representations of the two cue locations; in the Simon task, the task-irrelevant spatial locations where targets can be presented are either congruent or incongruent with the task-relevant response codes. Importantly, it is only the “Cued_Congruent” condition in which spatial IOR and the Simon effect may share the same spatial representation in the PPC, resulting in the observed behavioral response facilitation in that condition.

Our results were not consistent with the inhibitory theory. In that theory, increased Simon effect at the cued location is due to the incongruent condition responses being delayed at that inhibited location. Thus, we should have found higher neural activation in the “Cued_Incongruent” condition than in the “Cued_Congruent” condition, with the former conveying more conflict in it. Paradoxically, the bilateral middle frontal gyrus showed higher neural activity in the congruent than in the incongruent condition. In fact, one key difference between the classical Simon task and the one used in the present study is that the Simon stimuli were preceded by a spatial cue. Thus, once the attention orienting process, which shares the PPC neural network with the Simon effect, is evoked by the peripheral cue prior to the occurrence of the target, the attentional control set adopted by the bilateral middle frontal cortex might be matching the activated spatial representations with the response codes, in order to maximize the efficiency of behavioral responses. Therefore, whenever there was a match between the oriented spatial representations and the response codes, the bilateral frontal cortex caught it and showed higher neural activity (Figure 4B). That only occurs in the “Cued_Congruent” condition.

In line with the previous contention, the neural interaction contrast “Cued (Congruent > Incongruent) > Uncued (Congruent > Incongruent)” suggests that the left superior precentral gyrus was significantly activated by the neural interaction between spatial IOR and the Simon effect by showing significantly enhanced neural activity in the “Cued_Congruent” condition (Figure 5). Due to its topographic organization, the precentral gyrus (also known as the primary sensorimotor cortex) is traditionally considered the cortical area for voluntary movement (Ugur et al., 2005). More importantly, the superior region of the precentral gyrus is significantly involved in hand representation, object manipulation (Sastre-Janer et al., 1998; Boling et al., 1999; Rose et al., 2012) and motor execution (Stippich et al., 2002). Furthermore, the connectivity strength between the precentral and the postcentral gyrus is positively correlated with hand motor performance (Rose et al., 2012). In another fMRI study, shorter reaction times with finger button presses were found along with greater activation of the supplementary motor area and right frontal opercular cortex (Klöppel et al., 2007). These findings suggest that, higher neural activity in the premotor cortex may facilitate the behavioral response. In the present study, the left precentral gyrus showed higher neural activity in the “Cued_Congruent” condition, in correspondence with the facilitated behavioral responses observed in that condition. As it has been shown, it produced larger Simon effects at the cued than at the uncued location.

Taken together, by combining spatial IOR with the Simon task, we not only replicated the previous observation of larger Simon effects at the cued location of spatial IOR, but also revealed the neural mechanisms underlying this phenomenon. The key results were consistent with the shared spatial representation hypothesis. When the target appeared at the cued location and the cued location was congruent with the response code, the shared spatial representation system in the PPC between spatial IOR and the Simon effect was activated. Besides, the sensorimotor system in the precentral gyrus showed significantly enhanced neural activity, caused significant faster responses (i.e., a facilitatory effect) in the “Cued_Congruent” condition compared with the “Cued_Incongruent” condition, indirectly resulting in the increased size of the Simon effect observed at the cued location.

### Conflict of interest statement

The authors declare that the research was conducted in the absence of any commercial or financial relationships that could be construed as a potential conflict of interest.
